# Successful papillary large-balloon dilation using a novel nonslip balloon catheter in a patient with Roux-en-Y gastrectomy

**DOI:** 10.1055/a-2489-8393

**Published:** 2024-12-10

**Authors:** Yuki Tanisaka, Shomei Ryozawa, Masafumi Mizuide, Akashi Fujita, Ryuhei Jinushi, Ryuichi Watanabe, Ryo Sato

**Affiliations:** 1 183786 Gastroenterology, Saitama Medical University International Medical Center, Hidaka, Japan

Endoscopic papillary large-balloon dilation (EPLBD) 1 has been widely used to extract large stones from the bile duct in patients with Roux-en-Y gastrectomy, using a balloon enteroscope 2 3 . However, performing EPLBD with a balloon enteroscope is challenging owing to difficulty in securing a stable scope position and the lack of a forceps elevator, which can lead to balloon slippage during inflation. To overcome these limitations, a novel nonslip balloon catheter (RIGEL; Japan Lifeline, Japan) was developed. The distal and proximal ends of the balloon inflate first, followed by the middle, to prevent the catheter from slipping in or out of the papilla. Additionally, a black elastic band is attached to the center of the balloon to aid in endoscopic confirmation of the balloon's position. It has previously demonstrated effectiveness for balloon dilation with a balloon enteroscope using a balloon with an 8-mm diameter 4 5 . Recently, a balloon with a 12-mm diameter ( Fig. 1 ) has been added to the lineup to facilitate EPLBD with a balloon enteroscope. We report a case of successful EPLBD using a novel nonslip balloon catheter in a patient with Roux-en-Y gastrectomy.

**Fig. 1:**
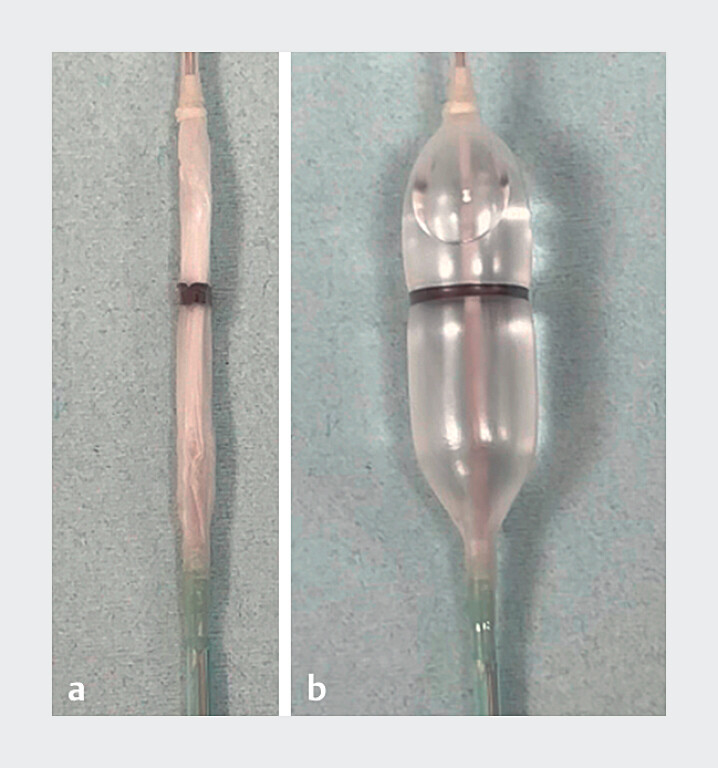
Photograph of the novel nonslip balloon catheter (RIGEL; Japan Lifeline, Japan) with a 12-mm diameter. The distal and proximal ends of the balloon initially inflate to prevent the catheter from slipping in or out of the papilla. A black elastic band is attached to the center of the balloon to aid in endoscopic confirmation of the balloonʼs position.

A 78-year-old woman presented with cholangitis due to choledocholithiasis. She had undergone Roux-en-Y gastrectomy owing to gastric cancer. Endoscopic retrograde cholangiopancreatography (ERCP) was performed using a short-type single-balloon enteroscope (SIF-H290; Olympus Marketing, Japan) with a working length of 152 cm and a working channel of 3.2 mm in diameter 2 ( Video 1 ). Cholangiography revealed an approximately 12-mm stone in the common bile duct ( Fig. 2 ). Subsequently, EPLBD was performed using a novel nonslip balloon catheter with a 12-mm diameter. EPLBD was performed effectively without slippage during inflation, successfully dilating the papilla ( Fig. 3 ). Finally, the stone was extracted smoothly using a basket catheter ( Fig. 4 ).

Successful papillary large-balloon dilation using a novel nonslip balloon catheter in a patient with Roux-en-Y gastrectomy.Video 1

**Fig. 2:**
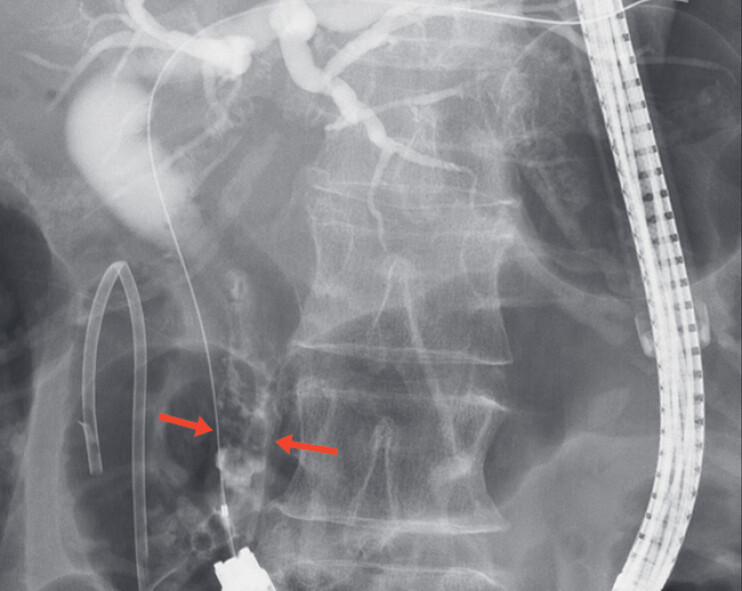
Cholangiographic image showing an approximately 12-mm stone (red arrows) in the common bile duct.

**Fig. 3:**
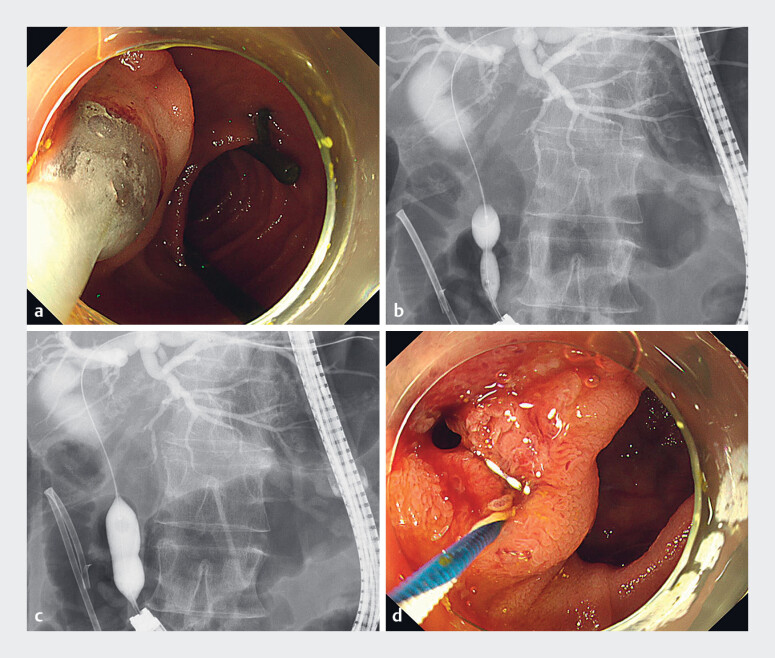
Endoscopic and cholangiographic images during endoscopic papillary large-balloon dilation (ELPBD) showing: **a** the balloon being inflated without slippage; **b, c** large-balloon dilation being effectively performed; **d** the dilated orifice of the papilla.

**Fig. 4:**
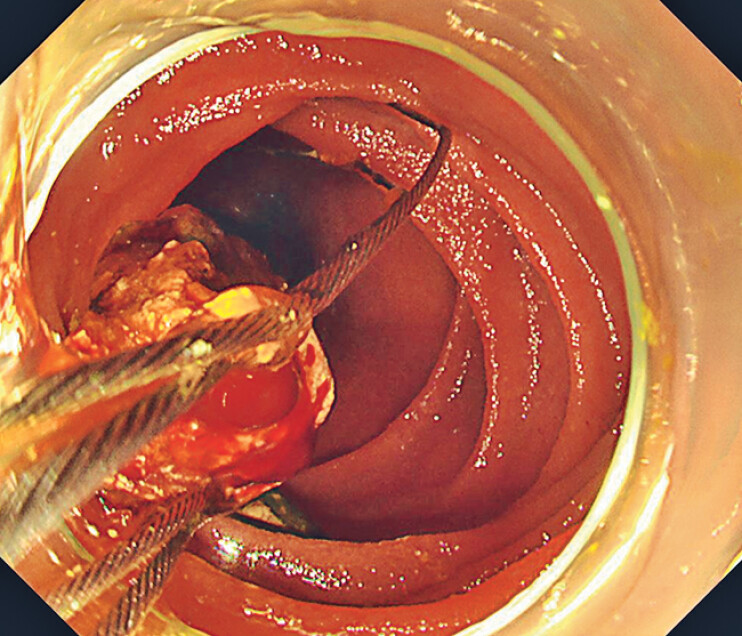
Endoscopic image showing successful stone extraction using a basket catheter.

This novel nonslip balloon catheter can facilitate EPLBD in patients with Roux-en-Y gastrectomy.

Endoscopy_UCTN_Code_TTT_1AR_2AC

## Notes

Endoscopy E-Videos https://eref.thieme.de/e-videos
*E-Videos* is an open access online section of the journal *Endoscopy* , reporting on interesting cases and new techniques in gastroenterological endoscopy. All papers include a high-quality video and are published with a Creative Commons CC-BY license. Endoscopy E-Videos qualify for HINARI discounts and waivers and eligibility is automatically checked during the submission process. We grant 100% waivers to articles whose corresponding authors are based in Group A countries and 50% waivers to those who are based in Group B countries as classified by Research4Life (see: https://www.research4life.org/access/eligibility/ ). This section has its own submission website at https://mc.manuscriptcentral.com/e-videos .
